# Etiology of trauma-related acute compartment syndrome of the forearm: a systematic review

**DOI:** 10.1186/s13018-022-03234-x

**Published:** 2022-07-06

**Authors:** Khalid I. Khoshhal, Ehab F. Alsaygh, Obaid F. Alsaedi, Alwaleed A. Alshahir, Ammar F. Alzahim, Mohammad S. Al Fehaid

**Affiliations:** 1 Department of Surgery, Prince Mohammed Bin Abdulaziz Hospital, Ministry of National Guard Health-Affairs, Almadinah Almunawwarah, Saudi Arabia; 2 College of Medicine, Taibah University, Almadinah Almunawwarah, Saudi Arabia; 3 College of Medicine, King Saud Bin Abdulaziz University for Health Sciences, Riyadh, Saudi Arabia; 4 College of Medicine, Imam Abdulrahman Bin Faisal University, Dammam, Saudi Arabia

**Keywords:** Acute compartment syndrome, Etiology, Fracture, Soft tissue injury, Trauma, Vascular injury

## Abstract

**Objectives:**

Acute compartment syndrome (ACS) can be caused by multiple causes that affect people of different ages. It is considered an orthopedic emergency condition that requires immediate diagnosis and surgical intervention to avoid devastating complications and irreversible damages. This systematic review aimed to present the etiology of trauma-related forearm ACS.

**Methods:**

A systematic review was performed on four different databases: Embase, Medline, Cochrane Central Register of Controlled Trials (CENTRAL) and Cochrane Database of systematic review register databases via Ovid, with no restriction on dates (last date was June 30, 2021). It included all the studies containing data about the etiology of trauma-related forearm ACS.

**Results:**

A total of 4893 articles were retrieved: 122 met the inclusion criteria, 39 were excluded, 25 were out of scope and 14 had insufficient details. Hence, this review constituted 83 articles and 684 patients. The etiology of ACS causing forearm ACS was classified into three groups: fracture-related, soft tissue injury-related and vascular injury-related. The fracture-related group was the most common group (65.4%), followed by soft tissue injury (30.7%), then vascular injuries (3.9%). Furthermore, supracondylar humerus fractures were the most common cause of fractures related to forearm ACS. Blunt traumas were the most common cause of soft tissue injuries-related forearm ACS, and brachial artery injuries were the most common cause of vascular-related forearm ACS.

**Conclusion:**

Frequent assessment of patients with the most prevalent etiologies of forearm ACS is recommended for early detection of forearm ACS and to save limbs.

## Introduction

Acute compartment syndrome (ACS) of the forearm is defined as increased pressure in the closed osteofascial compartment of the forearm with a compromised microcirculation leading to ischemia and tissue damage [1]. The myofascial compartments of the upper extremity are divided into the shoulder and arm, the forearm and the hand based on the anatomic region [2]. Forearm is the most common site for ACS in the upper extremity and the second most common site for ACS in the body after the leg [3]. The diagnosis of ACS is mainly clinical, with pain that is disproportionate to the size of injury, being a known hallmark component of the clinical presentation. However, the patient may not exhibit clinical signs, which makes this a challenging task for the on-call trauma surgeon to diagnose and eventually increase the risk of late or misdiagnosis of this pathology [4, 5]. Thus, a high index of clinical suspicion is needed when treating ACS. A delay in the treatment could lead to complications including neurological deficits, fracture nonunion, muscle necrosis, chronic pain, and forearm contractures [6–9].

To our knowledge, there have been no previous systematic reviews that have focused on the possible etiologies of acute trauma-related forearm compartment syndrome, except for the Kalyani et al. [6] study in 2011. They have discussed both traumatic and non-traumatic etiologies of ACS. In addition, various studies have been presented to the literature since then, which contain additional information about the etiologies of ACS. Finally, understanding the causes of this devastating condition allows us to categorize the risk by identifying the group of high-risk patients, and the most common traumatic etiologies associated with them, leading to early diagnosis and intervention, fewer complications and overall better outcome.

Therefore, the aim of this systematic review was to identify the most prevalent etiologies in the literature regarding acute trauma-related compartment syndrome of the forearm.

## Materials and methods

**Fig. 1:**
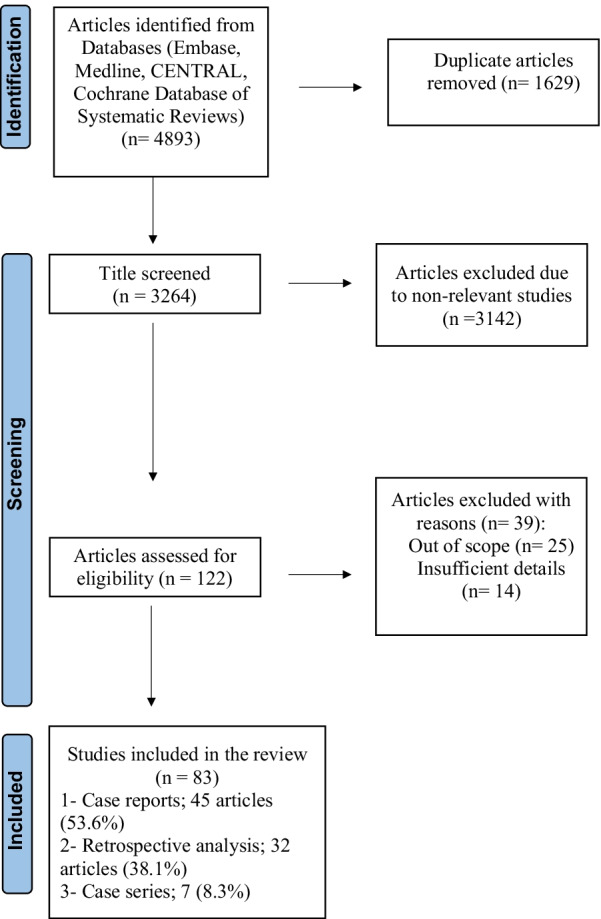
PRISMA flowchart

This systematic review was conducted and reported in light of the Preferred Reporting Items for Systematic Reviews and Meta-Analysis (PRISMA) checklist (Fig. 1).

### Eligibility criteria

The inclusion criteria were studies containing data about the etiology of trauma-related forearm ACS, with available full text. The exclusion criteria were animal studies and non-English studies.

### Search strategy

The Embase, Medline, Cochrane Central Register of Controlled Trials (CENTRAL) and Cochrane Database of systematic review register databases via Ovid were searched with no date restrictions, using the keywords: compartment syndrome AND forearm OR vascular OR trauma OR radius fracture OR forearm fracture OR ulna fracture OR humeral supracondylar fracture. The last date of the systematic search was on June 30, 2021. A search through the reference lists of the included studies was carried out for any potentially missed articles that fit the study criteria.

### Study selection and extraction of data

Six authors carried out the eligibility screening of the titles and abstracts followed by full-text assessment, and data extraction from eligible articles was carried out. Finally, any disagreements were settled via discussions or decisions of a third author.

## Results

A total of 4893 articles were retrieved, but only 122 met the inclusion criteria. Thirty-nine reports were excluded, 25 of them were out of scope and 14 articles had insufficient details. Thus, 83 articles with 684 patients [7, 10–91] constituted the basis of this review.

The etiology of the forearm ACS was classified into three groups according to the affected anatomical structure that caused the compartment syndrome: fracture-related, soft tissue injury-related and vascular injury-related causes.

## Fracture-related ACS

Fifty-one articles provided relevant data about 447 fractured patients who developed an ACS (65.4%) [7, 10–59]. Eleven articles identified supracondylar humerus fracture (SCF) as the most prevalent site of fracture leading up to 152 cases (34% of SCF patients) of fractured patients causing ACS [7, 10, 11, 14, 15, 19, 22, 26, 52, 57, 58]; 19 articles reported both-bone forearm fractures making up to 123 cases (27.5%) of fractured patients [10, 16, 17, 20, 26, 29, 33, 34, 40, 44, 45, 50–53, 55, 56, 58]. Twenty articles reported distal radial fractures leading to ACS in 99 cases (22.1%) of fractured patients (Table 1) [10, 12, 18, 24, 26, 29, 30, 32, 39, 41, 43, 46–49, 51–53, 58].

**Table 1:** Fracture-related acute compartment syndrome

N	Type of fracture	Number out of 447 fractured patients	Percentage out of 447 fractured patients
1	Supracondylar humerus fracture [7, 10, 11, 14, 15, 19, 22, 26, 52, 57, 58]	152	34.0
2	Both-bone forearm fracture [10, 16, 17, 20, 26, 29, 33, 34, 40, 44, 45, 50–53, 55, 56, 58]	123	27.5
3	Distal radial fractures [10, 12, 18, 24, 26, 29, 30, 32, 39, 41, 43, 45–49, 51–53, 58]	99	22.1
4	Forearm bone fracture without identification of fractured segment [21, 25, 27, 42, 51, 52]	16	3.6
5	Distal radius fracture with elbow fracture [29]	9	2.0
6	Proximal third of forearm [54]	8	1.8
7	Monteggia equivalents [11]	7	1.6
8	Supracondylar humerus fracture and both-bone forearm fracture [7, 26, 38]	7	1.6
9	Radial neck fractures [26, 36, 56, 58]	4	0.9
10	Unspecified radius fracture [16, 50]	4	0.9
11	Olecranon fracture [10, 27]	3	0.7
12	Monteggia fractures [11]	2	0.4
13	Radial shaft fracture [59]	2	0.4
14	Unspecified ulna fracture [16, 58]	2	0.4
15	Proximal ulna fracture [10]	1	0.2
16	Ulna diaphyseal fracture [40]	1	0.2
17	Distal ulna fracture [43]	1	0.2
18	Ulnar diaphyseal fracture and fracture of radius in distal one-fourth and dislocation of radial head [13]	1	0.2
19	Ulnar shaft fracture and supracondylar humerus fracture [23]	1	0.2
20	Supracondylar humerus fracture, ulnar shaft fracture and distal both-bone forearm fracture [26]	1	0.2
21	Anterior elbow dislocation with ulnar shaft and radial styloid fracture [31]	1	0.2
22	Distal radial fracture with basi-styloid fracture of the ulna [35]	1	0.2
23	Supracondylar humerus fracture and distal radius fracture [37]	1	0.2

## Soft tissue injury-related ACS

Thirty-seven articles observed 210 patients who developed ACS (30.7%) after a soft tissue injury [7, 50–58, 60–86]. Four articles identified blunt trauma in 44 patients as the most prevalent cause of soft tissue trauma-related ACS, leading up to 20.9% of soft tissue-injured patients [57, 82, 85, 86]. Three identified 39 patients with burns (18.6%) [61, 76, 85], and six reported 36 patients with ACS (17.1%) that occurred after crush injury [65, 73, 74, 80, 85, 86]. Two did not specify the extent of the trauma, reporting generic soft tissue injuries in 37 patients as the cause of ACS (Table 2) [51, 53].

**Table 2:** Soft tissue-related acute compartment syndrome

N	Name of soft tissue injury	Number out of 210 soft tissue injury patients	Percentage out of 210 soft tissue injury patients
1	Blunt trauma [57, 69, 75, 82, 85, 87]	44	20.9
2	Burn injuries [61, 76, 85]	39	18.5
3	Not reported by the authors [51, 53]	37	17.6
4	Crush injury [65, 73, 74, 80, 85, 86]	36	17
5	Penetrating trauma [7, 54, 73]	22	10.5
6	Forearm caught in a machine [50, 56, 58, 62, 66]	10	4.7
7	Snake bites [60, 63, 81]	4	1.9
8	Suction injuries [70, 72, 78]	4	1.9
9	Traction injury [67, 71]	2	1.0
10	Increased intrauterine pressure [79, 84]	2	1.0
11	Fall injuries [55, 77]	2	1.0
12	Spider bite [68]	1	0.5
13	Flying insect bite [64]	1	0.5
14	Compression trauma [79]	1	0.5
15	Electrical injury [50]	1	0.5
16	Caught by a wall [50]	1	0.5
17	Carving fork wound [7]	1	0.5
18	Dog bite [52]	1	0.5
19	Roller injury [86]	1	0.5

## Vascular injury-related ACS

Eight articles related ACS to vascular injuries in 27 patients (3.9%) [59, 85–91]. Two articles [86, 91] reported seven patients who developed ACS after brachial artery injuries in 25.9% of ACS cases secondary to vascular injury. Two reported two patients, each with ACS, developed after ulnar artery injury [59, 88], and three reported 13 cases of forearm ACS developed after unspecified vascular injuries (Table 3) [85, 89, 90].

**Table 3:** Vascular injury-related acute compartment syndrome

N	Name of the vessel	Number of patients out of 27 patients with vascular injury	Percentage out of 27 patients with vascular injury
1	Not reported by the authors [85, 89, 90]	13	48.1
2	Brachial artery [59, 91]	7	25.9
3	Radial artery [59]	3	11.1
4	Anterior interosseous artery [59, 87]	2	7.4
5	Ulnar artery [59, 88]	2	7.4

Within the 13 articles [7, 50–59, 85, 86] that were suitable to be included in two of the three groups, ten [7, 50–58] were included in fracture-related and soft tissue injury-related groups. In addition, one [59] was included in fracture-related and vascular injury-related groups, while the other two [85, 86] were included in vascular-related and soft tissue-related groups.

## Discussion

In the current systemic review, the etiologies of traumatic forearm compartment syndrome were evaluated, revealing that fractures were the most common etiology leading to forearm ACS, in particular supracondylar humerus fractures. In addition, soft tissue injuries (blunt trauma) following fractures showed higher rates of forearm ACS than any other soft tissue injuries. Finally, vascular injuries were the least to cause forearm ACS.

Furthermore, fractures were the most common etiology of forearm ACS (65.3%), revealing that supracondylar humerus fractures were the most prevalent sites of fractures, occurring in 34% of fracture cases causing ACS, followed by both-bone forearm fractures (27.5%), followed by distal radius fractures (22.1%). Though supracondylar fractures were the most prevalent fracture causing forearm ACS, this should be looked at in view of Robertson et al. [15] study that described an incidence of 0.2% of ACS in supracondylar humeral fractures (67 cases of ACS out of 31,234 supracondylar fracture cases). This shows that supracondylar fractures are a common cause of forearm ACS, but ACS is not that common in supracondylar fractures. In addition, supracondylar humerus fractures were the most prevalent in the current review, which could be attributable to the vast pediatric population in this study. In line with the results, fractures were also the most common cause leading to 70% of ACS cases in Stella et al.’s systemic review of 95 articles on leg ACS [4].

When comparing the results of this study (83 articles) to that of Kalyani et al. [6] (12 articles), fractures were the leading cause of ACS and supracondylar fractures were the predominant cause out of these fractures, whereas their findings revealed that fractures were the second most common cause (after soft tissue injuries with a 2% difference) of ACS, consisting of 31%. The distal radius fracture was the most common site of fracture, occurring in 14.3% of their fracture cases (32 cases). However, in Oliver et al. [92] systemic review of the outcomes of forearm fasciotomies, they included 142 forearm ACS cases, but unfortunately, the etiologies were not mentioned.

Results also reveal that ACS injuries relating to the radius were more common in the distal part of the bone or concurrent injuries involving the radius with another bone [16, 33, 52, 53]. Duckworth et al.’s [51] findings of their study of 90 patients revealed that 31 (34%) distal radius fracture cases and 27 (30%) both-bone forearm fracture cases constituted 64% of the ACS cases, which is in line with the findings of the study in hand. Moreover, in 2009, Hwang et al. [29] (1286 patients) concluded that a fifty times higher risk was found in patients with combined distal radius fracture and elbow injury than patients with only distal radius fractures.

Another finding to consider is that the second most common cause of forearm ACS is soft tissue injury: Increased pressure within the compartment is associated with any injury to the surroundings or inside the compartment leading to ACS [53]. This injury can be secondary to either minor or major injuries, which have been observed to be blunt trauma in this study. Similarly, Stella et al. reported that soft tissue injuries were the second most common cause of leg ACS [4], and crush injuries were the most common cause of soft tissue injuries-related ACS.

Moreover, Kalyani et al.[6] (80 cases) reported a prevalence of 33.3% forearm ACS due to soft tissue injury, making it the most common cause of ACS. The differences between this study and theirs could be attributed to the fact that our study only focused on traumatic etiologies. In contrast, their study included all possible etiologies of forearm ACS regardless they were traumatic or not. Furthermore, Zhang et al. [85] study of 130 patients who underwent forearm fasciotomies 66% (86 patients) resulted from soft tissue injuries. Meanwhile, Özkan et al. [61] reported 43 fasciotomies in their study of 35 patients (81%) who had forearm ACS due to soft tissue injuries, indicating that even minimal injuries to soft tissues could lead to compartment syndrome. Thus, it is important not to underestimate any injuries, as forearm ACS was noticed to be caused by snakes, insects and spider bites [6, 60, 63, 64, 68, 81].

Results also revealed that forearm vascular-related ACS was mainly associated with trauma that could penetrate the skin and cause damage to the underlying vessels. Brachial artery was the most frequently affected artery causing ACS. Also, an increased pressure within a confined compartment by bleeding and local edema was the mechanism by which arterial injuries lead to ACS. Morin et al. [59] (2009) reported 3.8% cases of forearm ACS (5 out of 129) that were attributed to vascular trauma secondary to penetrating trauma. Similarly, Lagerstrom et al. [91] reported 32 cases of ACS, where 9 were in the forearm and 5 (15.6%) were due to brachial artery injury. Furthermore, Kalyani et al. [6] reported cases of forearm ACS (10.7%) that were attributed to vascular injuries.

The age range of each etiology was 3–75 years for fracture-related ACS, 7 days–68 years for soft tissue-related ACS, and newborns–33 years for vascular-related ACS.

Numerous complications can arise as a consequence of forearm ACS, including but not limited to nerve damage, gangrene, Volkmann’s contracture and rhabdomyolysis. These complications can affect the patient’s life greatly [93]. Therefore, the findings of this review help raise the suspicion around the traumatic etiologies of forearm ACS and guides the on-call trauma surgeon in decision-making, early diagnosis and avoiding the dreadful outcomes of this condition.

The strength of this review is the diversity and number of cases, which gave it a better estimate of the general population, although one of the limitations of this review was the incomplete information in many of the articles reviewed. In addition, 45 studies (53.6%) were case reports and retrospective case series, 32 (38.1%) level IV evidence, and several studies were conducted before 20 years or more. Thus, the need for more studies discussing the etiology of forearm ACS in a multicenter and prospective setting is recommended. We also acknowledge that this review was limited to studies written in English.

## Conclusion

Forearm ACS can be caused by multiple etiologies that affect people of different ages. Therefore, identifying the most prevalent causes can help in early detection of ACS by frequent assessment of patients that present with the most prevalent causes like supracondylar humerus fractures among fractures and blunt trauma among soft tissue injuries.

## Data Availability

Data and materials are available upon request.
